# [*N*,*N*,*N*′,*N*′-Tetra­kis(benzimidazol-2-ylmeth­yl)cyclo­hexane-1,2-*trans*-diamine]­iron(II) bis­(perchlorate) methanol solvate

**DOI:** 10.1107/S1600536811011810

**Published:** 2011-04-07

**Authors:** Gui-Ling Wu, Chun-Ping Ou, Feng Wang, Jian-Ming Zhang, She-Min Lan

**Affiliations:** a Key Laboratory of Pesticide & Chemical Biology, Ministry of Education, College of Chemistry, Central China Normal University, Wuhan 430079, People’s Republic of China

## Abstract

In the title compound, [Fe(C_38_H_38_N_10_)](ClO_4_)_2_·CH_3_OH, the Fe^II^ atom has a distorted octa­hedral coordination environment with four benzimidazol N atoms and two amino N atoms from an *N*,*N*,*N*′,*N*′-tetra­kis­(benzimidazol-2-ylmeth­yl)cyclo­hexane-1,2-*trans*-diamine ligand. The uncoordinated solvent methanol mol­ecule is hydrogen bonded to an O atom of a perchlorate anion. One of the perchlorate anions is disordered over two sets of sites with occupancy factors of 0.539 (14) and 0.461 (14). N—H⋯O and C—H⋯O hydrogen bonds, as well as π–π stacking inter­actions between the imidazol rings and between the imidazol and benzene rings [centroid–centroid distances = 3.714 (2) and 3.705 (2) Å] give rise to a three-dimensional network.

## Related literature

For model systems containing pyrazole chelates and related groups, see: Main (1992 ▶). For iron complexes with *N*,*N*,*N*′,*N*′-tetra­kis­(2-benzimidazolylmeth­yl)cyclo­hexane-1,2-*trans*-diamine, see: Mei *et al.* (2010 ▶); Zhao *et al.* (2005 ▶). For the synthesis of the ligand, see: Hendriks *et al.* (1982 ▶).
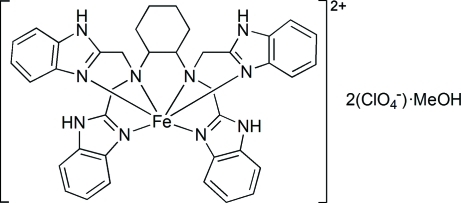


## Experimental

### 

#### Crystal data

[Fe(C_38_H_38_N_10_)](ClO_4_)_2_·CH_4_O
*M* *_r_* = 921.58
Monoclinic, 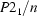

*a* = 14.3867 (6) Å
*b* = 15.9145 (6) Å
*c* = 18.8218 (8) Å
β = 103.175 (1)°
*V* = 4196.0 (3) Å^3^
*Z* = 4
Mo *K*α radiation
μ = 0.55 mm^−1^
*T* = 298 K
0.30 × 0.16 × 0.10 mm

#### Data collection

Bruker APEX CCD diffractometer
21311 measured reflections
7369 independent reflections
5765 reflections with *I* > 2σ(*I*)
*R*_int_ = 0.083

#### Refinement

*R*[*F* ^2^ > 2σ(*F* ^2^)] = 0.056
*wR*(*F* ^2^) = 0.176
*S* = 1.07
7369 reflections
612 parameters
10 restraints
H atoms treated by a mixture of independent and constrained refinement
Δρ_max_ = 0.57 e Å^−3^
Δρ_min_ = −0.53 e Å^−3^

### 

Data collection: *SMART* (Bruker, 2007 ▶); cell refinement: *SAINT* (Bruker, 2007 ▶); data reduction: *SAINT*; program(s) used to solve structure: *SHELXS97* (Sheldrick, 2008 ▶); program(s) used to refine structure: *SHELXL97* (Sheldrick, 2008 ▶); molecular graphics: *PLATON* (Spek, 2009 ▶); software used to prepare material for publication: *PLATON*.

## Supplementary Material

Crystal structure: contains datablocks global, I. DOI: 10.1107/S1600536811011810/hy2418sup1.cif

Structure factors: contains datablocks I. DOI: 10.1107/S1600536811011810/hy2418Isup2.hkl

Additional supplementary materials:  crystallographic information; 3D view; checkCIF report

## Figures and Tables

**Table 1:** Hydrogen-bond geometry (Å, °)

*D*—H⋯*A*	*D*—H	H⋯*A*	*D*⋯*A*	*D*—H⋯*A*
N4—H4*A*⋯O1^i^	0.86	2.10	2.952 (6)	171
N6—H6*A*⋯O6′^ii^	0.86	2.02	2.852 (15)	162
N6—H6*A*⋯O6^ii^	0.86	2.13	2.955 (16)	162
N8—H8*A*⋯O1*S*	0.86	1.94	2.790 (5)	170
N10—H10*A*⋯O7′^iii^	0.86	2.04	2.896 (10)	176
N10—H10*A*⋯O8^iii^	0.86	2.01	2.787 (12)	150
O1*S*—H1*S*⋯O3^iv^	0.96	1.88	2.813 (6)	166
C7—H7*B*⋯O4^i^	0.94 (4)	2.34 (4)	3.202 (6)	152 (3)
C23—H23*A*⋯O2^iv^	0.97	2.40	3.318 (6)	158

Symmetry codes: (i) 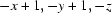; (ii) 
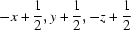
; (iii) 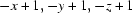; (iv) 
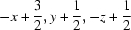
.

**Fig. 1.**
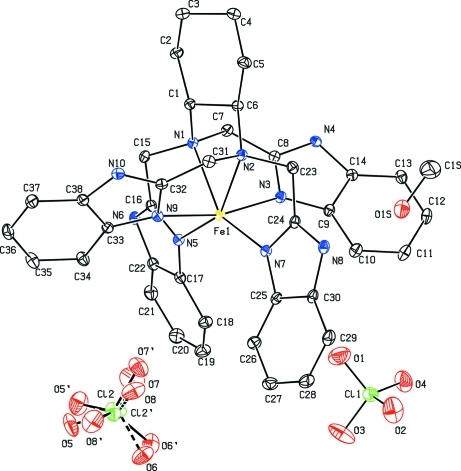
Molecular structure of the title compound. Displacement ellipsoids are drawn at the 30% probability level. H atoms have been omitted for clarity.

**Fig. 2.**
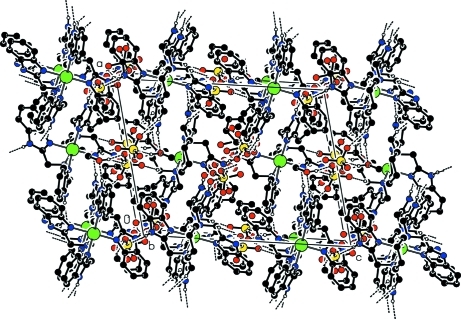
Crystal packing of the title compound, showing the formation of the three-dimensional network by hydrogen bonds and π–π interactions. H atoms not involved in the motif have been omitted for clarity.

## supplementary crystallographic information

### Comment

*N*,*N*,*N*',*N*'-Tetrakis(2-benzimidazolymethyl) cyclohexane-1,2-*trans*-diamine (ctb) is a benzimidazole-rich ligand, which has the advantage that the basicity of the coordinating group approximates that of histidine (histidine: pKb = 7.96; benzimidazole: pKb = 8.47; Main, 1992). The metal iron(III) and iron(II) can ligated by the ctb ligand, modeling the activities of lipoxygenase (Mei *et al.*, 2010; Zhao *et al.*, 2005). In a continuation of this work, herein we report the synthesis and structure of the title complex.

In the title compound, the Fe^II^ atom has a distorted octahedral coordination environment with four benzimidazol (bzim) N atoms and two amino N atoms from a ctb ligand (Fig. 1). The two *trans*-tertiary amine N atoms are significantly farther away from the Fe^II^ atom than the four bzim N atoms [average Fe—N_amine_ = 2.325 (3), Fe—N_bzim_ = 2.128 (3) Å]. As shown in Fig. 2, The uncoordinated solvent methanol molecule is hydrogen bonded to an O atom of a perchlorate anion. N—H···O and C—H···O hydrogen bonds, as well as π–π stacking interactions between the imidazol rings and between the imidazol and benzene rings [centroid–centroid distances = 3.714 (2) and 3.705 (2) Å] give rise to a three-dimensional network.

### Experimental

All reagents and solvents were used as obtained without further purification. The ctb ligand was prepared according to literature methods (Hendriks *et al.*, 1982). Equimolar amounts of ctb (63.4 mg, 0.10 mmol) and Fe(ClO_4_)_2_.6H_2_O (36.3 mg, 0.10 mmol) were dissolved in 25 ml MeOH, and the solution was stirred for 10 min. After filtration, colorless crystals of the title compound (yield: 79 mg, 85% ) were obtained by diffusion of diethyl ether into the filtrate after one week under Ar atmosphere.

### Refinement

One of the perchlorate anions is disordered over two positions and the command '*DFIX*' was used in the refinements. The final most satisfactory occupancies for the major and minor components were 0.539 (14) and 0.461 (14), respectively.

C-bound H atoms were positioned geometrically and refined as riding atoms, with C—H = 0.93 (aromatic), 0.97 (methylene) and 0.96 (methyl) Å and *U*_iso_(H) = 1.2(1.5 for methyl)*U*_eq_(C). H atoms attached to C2 and C7 were refined isotropically. N- and O-bound H atoms were first found in difference Fourier maps and then placed at their ideal postions, with N—H = 0.86 and O—H = 0.82 Å and *U*_iso_(H) = 1.2*U*_eq_(N) or 1.5*U*_eq_(O).

### Crystal data

**Table:**

[Fe(C_38_H_38_N_10_)](ClO_4_)_2_·CH_4_O	*F*(000) = 1912
*M**_r_* = 921.58	*D*_x_ = 1.459 Mg m^−^^3^
Monoclinic, *P*2_1_/*n*	Mo *K*α radiation, λ = 0.71073 Å
Hall symbol: -P 2yn	Cell parameters from 8487 reflections
*a* = 14.3867 (6) Å	θ = 2.4–28.0°
*b* = 15.9145 (6) Å	µ = 0.55 mm^−^^1^
*c* = 18.8218 (8) Å	*T* = 298 K
β = 103.175 (1)°	Block, colorless
*V* = 4196.0 (3) Å^3^	0.30 × 0.16 × 0.10 mm
*Z* = 4	

### Data collection

**Table:**

Bruker APEX CCD diffractometer	5765 reflections with *I* > 2σ(*I*)
Radiation source: fine-focus sealed tube	*R*_int_ = 0.083
graphite	θ_max_ = 25.0°, θ_min_ = 2.0°
φ and ω scans	*h* = −15→17
21311 measured reflections	*k* = −17→18
7369 independent reflections	*l* = −22→22

### Refinement

**Table:**

Refinement on *F*^2^	Primary atom site location: structure-invariant direct methods
Least-squares matrix: full	Secondary atom site location: difference Fourier map
*R*[*F*^2^ > 2σ(*F*^2^)] = 0.056	Hydrogen site location: inferred from neighbouring sites
*wR*(*F*^2^) = 0.176	H atoms treated by a mixture of independent and constrained refinement
*S* = 1.07	*w* = 1/[σ^2^(*F*_o_^2^) + (0.1022*P*)^2^ + 2.0578*P*] where *P* = (*F*_o_^2^ + 2*F*_c_^2^)/3
7369 reflections	(Δ/σ)_max_ < 0.001
612 parameters	Δρ_max_ = 0.57 e Å^−^^3^
10 restraints	Δρ_min_ = −0.53 e Å^−^^3^

### Fractional atomic coordinates and isotropic or equivalent isotropic displacement parameters (Å^2^)

**Table:**

	*x*	*y*	*z*	*U*_iso_*/*U*_eq_	Occ. (<1)
Fe1	0.49217 (3)	0.51266 (3)	0.25944 (3)	0.03221 (18)	
N1	0.40904 (18)	0.63557 (16)	0.22008 (16)	0.0340 (6)	
N2	0.59753 (19)	0.61865 (17)	0.31077 (17)	0.0367 (7)	
N3	0.5240 (2)	0.53017 (17)	0.15388 (17)	0.0387 (7)	
N4	0.5194 (2)	0.61519 (17)	0.06081 (16)	0.0378 (7)	
H4A	0.5027	0.6569	0.0317	0.045*	
N5	0.35367 (19)	0.46902 (17)	0.21018 (16)	0.0357 (6)	
N6	0.2082 (2)	0.50488 (18)	0.15204 (17)	0.0399 (7)	
H6A	0.1577	0.5350	0.1380	0.048*	
N7	0.62225 (19)	0.45068 (17)	0.29869 (16)	0.0370 (7)	
N8	0.7795 (2)	0.4561 (2)	0.33400 (18)	0.0455 (8)	
H8A	0.8364	0.4764	0.3434	0.055*	
N9	0.4597 (2)	0.52468 (17)	0.36472 (17)	0.0389 (7)	
N10	0.4732 (2)	0.60837 (17)	0.45995 (16)	0.0418 (7)	
H10A	0.4973	0.6451	0.4925	0.050*	
C1	0.4544 (2)	0.7063 (2)	0.2673 (2)	0.0377 (8)	
H1A	0.4429	0.6957	0.3159	0.045*	
C2	0.4124 (3)	0.7932 (2)	0.2440 (3)	0.0510 (10)	
H2A	0.348 (3)	0.795 (3)	0.251 (3)	0.066 (13)*	
H2B	0.407 (3)	0.800 (3)	0.196 (3)	0.061 (14)*	
C3	0.4708 (3)	0.8639 (2)	0.2853 (3)	0.0591 (11)	
H3A	0.4433	0.9174	0.2666	0.071*	
H3B	0.4694	0.8605	0.3365	0.071*	
C4	0.5723 (3)	0.8592 (2)	0.2776 (3)	0.0580 (11)	
H4B	0.6082	0.9065	0.3024	0.070*	
H4C	0.5739	0.8622	0.2264	0.070*	
C5	0.6177 (3)	0.7774 (2)	0.3101 (3)	0.0535 (10)	
H5A	0.6826	0.7746	0.3037	0.064*	
H5B	0.6203	0.7770	0.3620	0.064*	
C6	0.5629 (2)	0.7006 (2)	0.2751 (2)	0.0379 (8)	
H6B	0.5729	0.6976	0.2254	0.045*	
C7	0.4096 (3)	0.6477 (2)	0.1418 (2)	0.0396 (8)	
H7A	0.350 (2)	0.6305 (19)	0.1137 (19)	0.040 (10)*	
H7B	0.416 (2)	0.705 (2)	0.131 (2)	0.040 (10)*	
C8	0.4854 (2)	0.5975 (2)	0.11997 (19)	0.0349 (7)	
C9	0.5883 (2)	0.5004 (2)	0.1148 (2)	0.0389 (8)	
C10	0.6475 (3)	0.4303 (2)	0.1245 (2)	0.0509 (10)	
H10B	0.6492	0.3930	0.1628	0.061*	
C11	0.7036 (3)	0.4184 (3)	0.0751 (2)	0.0577 (11)	
H11A	0.7429	0.3714	0.0797	0.069*	
C12	0.7031 (3)	0.4747 (3)	0.0187 (2)	0.0533 (10)	
H12A	0.7443	0.4656	−0.0121	0.064*	
C13	0.6443 (3)	0.5431 (2)	0.0068 (2)	0.0466 (9)	
H13A	0.6437	0.5801	−0.0316	0.056*	
C14	0.5858 (2)	0.5544 (2)	0.0551 (2)	0.0366 (8)	
C15	0.3110 (2)	0.6161 (2)	0.2272 (2)	0.0386 (8)	
H15A	0.2659	0.6559	0.1994	0.046*	
H15B	0.3071	0.6187	0.2779	0.046*	
C16	0.2893 (2)	0.5288 (2)	0.1979 (2)	0.0369 (8)	
C17	0.3114 (2)	0.4004 (2)	0.1689 (2)	0.0385 (8)	
C18	0.3487 (3)	0.3210 (2)	0.1593 (2)	0.0522 (10)	
H18A	0.4093	0.3049	0.1848	0.063*	
C19	0.2916 (3)	0.2680 (3)	0.1104 (3)	0.0653 (12)	
H19A	0.3138	0.2146	0.1028	0.078*	
C20	0.2023 (3)	0.2922 (3)	0.0723 (3)	0.0657 (13)	
H20A	0.1666	0.2550	0.0388	0.079*	
C21	0.1638 (3)	0.3692 (3)	0.0818 (2)	0.0561 (11)	
H21A	0.1030	0.3845	0.0562	0.067*	
C22	0.2201 (2)	0.4226 (2)	0.1312 (2)	0.0427 (9)	
C23	0.6903 (2)	0.5920 (2)	0.2965 (2)	0.0427 (8)	
H23A	0.7424	0.6207	0.3293	0.051*	
H23B	0.6928	0.6053	0.2467	0.051*	
C24	0.6979 (2)	0.4993 (2)	0.3088 (2)	0.0398 (8)	
C25	0.6562 (3)	0.3702 (2)	0.3196 (2)	0.0391 (8)	
C26	0.6065 (3)	0.2960 (2)	0.3245 (2)	0.0521 (10)	
H26A	0.5403	0.2936	0.3102	0.063*	
C27	0.6593 (3)	0.2266 (3)	0.3514 (3)	0.0649 (12)	
H27A	0.6284	0.1760	0.3549	0.078*	
C28	0.7582 (4)	0.2303 (3)	0.3735 (3)	0.0711 (14)	
H28A	0.7916	0.1822	0.3923	0.085*	
C29	0.8079 (3)	0.3027 (3)	0.3685 (3)	0.0607 (12)	
H29A	0.8741	0.3045	0.3824	0.073*	
C30	0.7551 (3)	0.3730 (2)	0.3420 (2)	0.0438 (9)	
C31	0.6018 (2)	0.6166 (2)	0.3898 (2)	0.0409 (8)	
H31A	0.6125	0.6730	0.4096	0.049*	
H31B	0.6549	0.5817	0.4140	0.049*	
C32	0.5113 (2)	0.5826 (2)	0.4044 (2)	0.0379 (8)	
C33	0.3809 (3)	0.5110 (2)	0.3957 (2)	0.0398 (8)	
C34	0.3042 (3)	0.4574 (3)	0.3759 (2)	0.0539 (10)	
H34A	0.2993	0.4199	0.3374	0.065*	
C35	0.2348 (3)	0.4618 (3)	0.4160 (3)	0.0653 (12)	
H35A	0.1823	0.4261	0.4043	0.078*	
C36	0.2417 (3)	0.5179 (3)	0.4731 (3)	0.0636 (12)	
H36A	0.1923	0.5203	0.4975	0.076*	
C37	0.3185 (3)	0.5701 (3)	0.4948 (2)	0.0542 (10)	
H37A	0.3234	0.6067	0.5340	0.065*	
C38	0.3891 (3)	0.5654 (2)	0.4551 (2)	0.0407 (8)	
Cl1	0.55488 (10)	0.17228 (8)	0.01931 (8)	0.0758 (4)	
O1	0.5176 (5)	0.2430 (3)	0.0429 (3)	0.141 (2)	
O2	0.6396 (4)	0.1476 (4)	0.0605 (3)	0.153 (2)	
O3	0.4884 (4)	0.1073 (3)	0.0204 (3)	0.155 (2)	
O4	0.5571 (4)	0.1862 (3)	−0.0548 (3)	0.1265 (18)	
Cl2	0.4081 (5)	0.1971 (4)	0.4315 (5)	0.064 (2)	0.461 (14)
O5	0.3219 (10)	0.1788 (13)	0.4499 (11)	0.124 (7)	0.461 (14)
O6	0.4447 (13)	0.1189 (8)	0.4236 (10)	0.113 (7)	0.461 (14)
O7	0.3795 (11)	0.2458 (9)	0.3656 (7)	0.164 (7)	0.461 (14)
O8	0.4841 (6)	0.2349 (7)	0.4747 (8)	0.107 (6)	0.461 (14)
Cl2'	0.4056 (7)	0.1978 (6)	0.4416 (6)	0.088 (3)	0.539 (14)
O5'	0.3123 (10)	0.2126 (13)	0.4188 (11)	0.132 (6)	0.539 (14)
O6'	0.4301 (11)	0.1350 (10)	0.3880 (10)	0.111 (6)	0.539 (14)
O7'	0.4525 (10)	0.2711 (7)	0.4260 (7)	0.119 (5)	0.539 (14)
O8'	0.4341 (9)	0.1893 (8)	0.5185 (5)	0.132 (5)	0.539 (14)
O1S	0.9648 (3)	0.5167 (3)	0.3479 (3)	0.0902 (12)	
H1S	0.9747	0.5408	0.3956	0.135*	
C1S	0.9867 (6)	0.5786 (5)	0.3004 (5)	0.137 (3)	
H1SA	1.0452	0.5637	0.2872	0.205*	
H1SB	0.9942	0.6323	0.3242	0.205*	
H1SC	0.9367	0.5828	0.2570	0.205*	

### Atomic displacement parameters (Å^2^)

**Table:**

	*U*^11^	*U*^22^	*U*^33^	*U*^12^	*U*^13^	*U*^23^
Fe1	0.0279 (3)	0.0359 (3)	0.0307 (3)	0.00317 (18)	0.0022 (2)	0.00087 (19)
N1	0.0307 (14)	0.0382 (14)	0.0321 (16)	0.0003 (11)	0.0053 (12)	−0.0031 (12)
N2	0.0301 (14)	0.0376 (14)	0.0403 (18)	0.0020 (11)	0.0034 (13)	0.0022 (13)
N3	0.0371 (15)	0.0428 (15)	0.0354 (18)	0.0078 (13)	0.0064 (13)	0.0030 (13)
N4	0.0402 (16)	0.0375 (15)	0.0351 (18)	−0.0002 (12)	0.0074 (14)	0.0041 (12)
N5	0.0322 (14)	0.0384 (14)	0.0338 (17)	0.0007 (12)	0.0023 (13)	−0.0014 (12)
N6	0.0278 (14)	0.0522 (17)	0.0384 (19)	0.0019 (12)	0.0045 (13)	0.0007 (14)
N7	0.0332 (15)	0.0420 (15)	0.0336 (17)	0.0058 (12)	0.0029 (13)	0.0011 (13)
N8	0.0302 (15)	0.0588 (18)	0.046 (2)	0.0082 (14)	0.0065 (14)	0.0031 (15)
N9	0.0390 (16)	0.0411 (15)	0.0359 (18)	−0.0031 (13)	0.0072 (14)	−0.0012 (13)
N10	0.0536 (18)	0.0399 (15)	0.0297 (18)	−0.0009 (14)	0.0050 (15)	−0.0032 (13)
C1	0.0372 (18)	0.0363 (17)	0.040 (2)	0.0013 (14)	0.0088 (16)	0.0012 (15)
C2	0.052 (2)	0.040 (2)	0.061 (3)	0.0091 (17)	0.013 (2)	0.0029 (19)
C3	0.068 (3)	0.0371 (19)	0.077 (3)	0.0017 (18)	0.025 (2)	−0.001 (2)
C4	0.064 (3)	0.040 (2)	0.074 (3)	−0.0087 (18)	0.024 (2)	−0.001 (2)
C5	0.050 (2)	0.043 (2)	0.066 (3)	−0.0102 (17)	0.009 (2)	−0.0019 (19)
C6	0.0385 (18)	0.0369 (17)	0.037 (2)	0.0002 (14)	0.0065 (16)	0.0009 (15)
C7	0.041 (2)	0.044 (2)	0.031 (2)	0.0080 (16)	0.0022 (17)	0.0026 (16)
C8	0.0329 (17)	0.0390 (17)	0.0307 (19)	0.0000 (14)	0.0029 (15)	−0.0016 (14)
C9	0.0328 (18)	0.0439 (18)	0.039 (2)	0.0017 (14)	0.0059 (16)	−0.0040 (16)
C10	0.054 (2)	0.059 (2)	0.038 (2)	0.0169 (19)	0.0064 (19)	0.0040 (18)
C11	0.053 (2)	0.072 (3)	0.047 (3)	0.023 (2)	0.009 (2)	−0.007 (2)
C12	0.044 (2)	0.076 (3)	0.042 (2)	0.004 (2)	0.0161 (19)	−0.013 (2)
C13	0.046 (2)	0.057 (2)	0.037 (2)	−0.0071 (18)	0.0110 (18)	−0.0047 (18)
C14	0.0327 (17)	0.0410 (18)	0.034 (2)	−0.0061 (14)	0.0032 (15)	−0.0052 (15)
C15	0.0314 (17)	0.0427 (18)	0.041 (2)	0.0066 (14)	0.0066 (16)	−0.0005 (15)
C16	0.0310 (17)	0.0441 (18)	0.035 (2)	−0.0021 (14)	0.0064 (16)	0.0010 (15)
C17	0.0389 (18)	0.0442 (18)	0.032 (2)	−0.0054 (15)	0.0076 (16)	−0.0009 (15)
C18	0.050 (2)	0.046 (2)	0.056 (3)	0.0009 (18)	0.004 (2)	−0.0037 (19)
C19	0.074 (3)	0.049 (2)	0.072 (3)	−0.006 (2)	0.012 (3)	−0.015 (2)
C20	0.061 (3)	0.065 (3)	0.067 (3)	−0.018 (2)	0.008 (2)	−0.023 (2)
C21	0.040 (2)	0.068 (3)	0.057 (3)	−0.0132 (19)	0.003 (2)	−0.009 (2)
C22	0.0330 (18)	0.049 (2)	0.045 (2)	−0.0049 (15)	0.0084 (17)	0.0006 (17)
C23	0.0318 (17)	0.050 (2)	0.045 (2)	−0.0024 (15)	0.0053 (16)	0.0020 (17)
C24	0.0324 (18)	0.050 (2)	0.036 (2)	0.0072 (15)	0.0068 (16)	0.0013 (16)
C25	0.0437 (19)	0.0438 (18)	0.030 (2)	0.0102 (15)	0.0083 (16)	0.0006 (15)
C26	0.052 (2)	0.051 (2)	0.050 (3)	0.0073 (18)	0.006 (2)	0.0052 (19)
C27	0.076 (3)	0.049 (2)	0.067 (3)	0.009 (2)	0.013 (3)	0.014 (2)
C28	0.072 (3)	0.061 (3)	0.080 (4)	0.031 (2)	0.017 (3)	0.023 (2)
C29	0.047 (2)	0.078 (3)	0.055 (3)	0.025 (2)	0.009 (2)	0.014 (2)
C30	0.042 (2)	0.056 (2)	0.034 (2)	0.0174 (17)	0.0107 (17)	0.0063 (17)
C31	0.0411 (19)	0.0462 (19)	0.031 (2)	−0.0019 (15)	−0.0010 (16)	−0.0008 (15)
C32	0.0377 (18)	0.0348 (17)	0.037 (2)	0.0020 (14)	0.0002 (16)	0.0012 (15)
C33	0.043 (2)	0.0390 (17)	0.038 (2)	0.0014 (15)	0.0102 (17)	0.0038 (15)
C34	0.058 (2)	0.062 (2)	0.043 (2)	−0.014 (2)	0.016 (2)	−0.003 (2)
C35	0.061 (3)	0.080 (3)	0.058 (3)	−0.020 (2)	0.020 (2)	0.002 (2)
C36	0.060 (3)	0.080 (3)	0.057 (3)	0.000 (2)	0.029 (2)	0.012 (2)
C37	0.073 (3)	0.058 (2)	0.036 (2)	0.009 (2)	0.021 (2)	0.0058 (18)
C38	0.051 (2)	0.0412 (18)	0.030 (2)	0.0043 (16)	0.0090 (17)	0.0067 (15)
Cl1	0.0785 (8)	0.0682 (7)	0.0822 (10)	0.0197 (6)	0.0213 (7)	0.0329 (6)
O1	0.202 (6)	0.101 (3)	0.129 (4)	0.045 (4)	0.054 (4)	0.023 (3)
O2	0.094 (3)	0.226 (6)	0.129 (5)	0.047 (4)	0.005 (3)	0.070 (4)
O3	0.178 (5)	0.140 (4)	0.133 (5)	−0.067 (4)	0.004 (4)	0.063 (4)
O4	0.127 (4)	0.156 (4)	0.107 (4)	0.032 (3)	0.049 (3)	0.068 (3)
Cl2	0.032 (3)	0.045 (3)	0.104 (6)	−0.0110 (19)	−0.007 (3)	−0.011 (3)
O5	0.092 (12)	0.167 (17)	0.130 (16)	−0.052 (10)	0.058 (11)	−0.013 (10)
O6	0.088 (8)	0.090 (8)	0.142 (18)	0.001 (6)	−0.012 (10)	−0.056 (10)
O7	0.157 (13)	0.176 (14)	0.145 (14)	−0.023 (11)	0.002 (11)	0.020 (11)
O8	0.077 (6)	0.082 (7)	0.130 (13)	−0.008 (5)	−0.044 (7)	−0.048 (8)
Cl2'	0.073 (4)	0.110 (5)	0.081 (3)	−0.029 (3)	0.021 (3)	−0.032 (3)
O5'	0.071 (7)	0.172 (16)	0.143 (16)	0.004 (8)	0.006 (8)	−0.025 (11)
O6'	0.083 (8)	0.127 (10)	0.128 (14)	−0.045 (7)	0.032 (8)	−0.065 (8)
O7'	0.148 (12)	0.114 (8)	0.105 (9)	−0.065 (8)	0.048 (9)	−0.040 (6)
O8'	0.141 (10)	0.160 (10)	0.084 (7)	0.031 (8)	0.005 (6)	0.006 (6)
O1S	0.074 (2)	0.099 (3)	0.098 (3)	−0.019 (2)	0.023 (2)	−0.040 (2)
C1S	0.177 (8)	0.099 (5)	0.146 (8)	−0.022 (5)	0.062 (7)	−0.028 (5)

### Geometric parameters (Å, °)

**Table:**

Fe1—N7	2.095 (3)	C13—C14	1.384 (5)
Fe1—N5	2.114 (3)	C13—H13A	0.9300
Fe1—N9	2.145 (3)	C15—C16	1.500 (5)
Fe1—N3	2.156 (3)	C15—H15A	0.9700
Fe1—N1	2.324 (3)	C15—H15B	0.9700
Fe1—N2	2.326 (3)	C17—C22	1.388 (5)
N1—C15	1.479 (4)	C17—C18	1.401 (5)
N1—C7	1.488 (4)	C18—C19	1.374 (6)
N1—C1	1.489 (4)	C18—H18A	0.9300
N2—C31	1.475 (5)	C19—C20	1.376 (6)
N2—C23	1.482 (4)	C19—H19A	0.9300
N2—C6	1.499 (4)	C20—C21	1.372 (6)
N3—C8	1.305 (4)	C20—H20A	0.9300
N3—C9	1.390 (4)	C21—C22	1.379 (5)
N4—C8	1.344 (4)	C21—H21A	0.9300
N4—C14	1.382 (4)	C23—C24	1.495 (5)
N4—H4A	0.8600	C23—H23A	0.9700
N5—C16	1.312 (4)	C23—H23B	0.9700
N5—C17	1.397 (4)	C25—C30	1.389 (5)
N6—C16	1.339 (4)	C25—C26	1.394 (5)
N6—C22	1.389 (4)	C26—C27	1.369 (6)
N6—H6A	0.8600	C26—H26A	0.9300
N7—C24	1.314 (5)	C27—C28	1.389 (7)
N7—C25	1.396 (4)	C27—H27A	0.9300
N8—C24	1.350 (4)	C28—C29	1.371 (6)
N8—C30	1.385 (5)	C28—H28A	0.9300
N8—H8A	0.8600	C29—C30	1.380 (5)
N9—C32	1.305 (4)	C29—H29A	0.9300
N9—C33	1.405 (4)	C31—C32	1.493 (5)
N10—C32	1.350 (5)	C31—H31A	0.9700
N10—C38	1.375 (5)	C31—H31B	0.9700
N10—H10A	0.8600	C33—C34	1.376 (5)
C1—C2	1.532 (5)	C33—C38	1.398 (5)
C1—C6	1.537 (5)	C34—C35	1.383 (6)
C1—H1A	0.9800	C34—H34A	0.9300
C2—C3	1.510 (6)	C35—C36	1.383 (7)
C2—H2A	0.97 (5)	C35—H35A	0.9300
C2—H2B	0.90 (5)	C36—C37	1.369 (6)
C3—C4	1.503 (6)	C36—H36A	0.9300
C3—H3A	0.9700	C37—C38	1.394 (5)
C3—H3B	0.9700	C37—H37A	0.9300
C4—C5	1.521 (6)	Cl1—O2	1.346 (5)
C4—H4B	0.9700	Cl1—O1	1.364 (5)
C4—H4C	0.9700	Cl1—O3	1.413 (5)
C5—C6	1.521 (5)	Cl1—O4	1.421 (5)
C5—H5A	0.9700	Cl2—O8	1.347 (8)
C5—H5B	0.9700	Cl2—O6	1.373 (9)
C6—H6B	0.9800	Cl2—O5	1.392 (9)
C7—C8	1.483 (5)	Cl2—O7	1.441 (9)
C7—H7A	0.94 (3)	Cl2'—O5'	1.333 (15)
C7—H7B	0.94 (4)	Cl2'—O7'	1.411 (12)
C9—C10	1.390 (5)	Cl2'—O8'	1.418 (14)
C9—C14	1.409 (5)	Cl2'—O6'	1.517 (15)
C10—C11	1.375 (6)	O1S—C1S	1.414 (8)
C10—H10B	0.9300	O1S—H1S	0.9556
C11—C12	1.389 (6)	C1S—H1SA	0.9600
C11—H11A	0.9300	C1S—H1SB	0.9600
C12—C13	1.365 (6)	C1S—H1SC	0.9600
C12—H12A	0.9300		
			
N7—Fe1—N5	132.58 (11)	C14—C13—H13A	121.8
N7—Fe1—N9	94.65 (11)	N4—C14—C13	132.6 (3)
N5—Fe1—N9	93.02 (11)	N4—C14—C9	104.9 (3)
N7—Fe1—N3	91.46 (11)	C13—C14—C9	122.4 (3)
N5—Fe1—N3	90.87 (11)	N1—C15—C16	106.5 (3)
N9—Fe1—N3	167.46 (11)	N1—C15—H15A	110.4
N7—Fe1—N1	149.54 (10)	C16—C15—H15A	110.4
N5—Fe1—N1	76.80 (10)	N1—C15—H15B	110.4
N9—Fe1—N1	90.85 (10)	C16—C15—H15B	110.4
N3—Fe1—N1	78.43 (10)	H15A—C15—H15B	108.6
N7—Fe1—N2	75.51 (10)	N5—C16—N6	112.6 (3)
N5—Fe1—N2	151.68 (10)	N5—C16—C15	121.8 (3)
N9—Fe1—N2	79.05 (11)	N6—C16—C15	125.3 (3)
N3—Fe1—N2	91.94 (11)	C22—C17—N5	109.1 (3)
N1—Fe1—N2	76.19 (10)	C22—C17—C18	120.7 (3)
C15—N1—C7	109.7 (3)	N5—C17—C18	130.2 (3)
C15—N1—C1	113.7 (3)	C19—C18—C17	116.8 (4)
C7—N1—C1	113.1 (3)	C19—C18—H18A	121.6
C15—N1—Fe1	103.09 (19)	C17—C18—H18A	121.6
C7—N1—Fe1	107.84 (19)	C18—C19—C20	121.4 (4)
C1—N1—Fe1	108.79 (19)	C18—C19—H19A	119.3
C31—N2—C23	110.2 (3)	C20—C19—H19A	119.3
C31—N2—C6	113.8 (3)	C21—C20—C19	122.6 (4)
C23—N2—C6	112.9 (3)	C21—C20—H20A	118.7
C31—N2—Fe1	106.03 (19)	C19—C20—H20A	118.7
C23—N2—Fe1	104.2 (2)	C20—C21—C22	116.4 (4)
C6—N2—Fe1	109.06 (19)	C20—C21—H21A	121.8
C8—N3—C9	106.4 (3)	C22—C21—H21A	121.8
C8—N3—Fe1	113.6 (2)	C21—C22—C17	122.0 (4)
C9—N3—Fe1	138.6 (2)	C21—C22—N6	132.9 (4)
C8—N4—C14	107.9 (3)	C17—C22—N6	105.0 (3)
C8—N4—H4A	126.1	N2—C23—C24	107.0 (3)
C14—N4—H4A	126.1	N2—C23—H23A	110.3
C16—N5—C17	105.6 (3)	C24—C23—H23A	110.3
C16—N5—Fe1	113.3 (2)	N2—C23—H23B	110.3
C17—N5—Fe1	138.4 (2)	C24—C23—H23B	110.3
C16—N6—C22	107.7 (3)	H23A—C23—H23B	108.6
C16—N6—H6A	126.1	N7—C24—N8	112.2 (3)
C22—N6—H6A	126.1	N7—C24—C23	122.0 (3)
C24—N7—C25	105.9 (3)	N8—C24—C23	125.8 (3)
C24—N7—Fe1	114.7 (2)	C30—C25—C26	120.8 (3)
C25—N7—Fe1	139.3 (2)	C30—C25—N7	108.9 (3)
C24—N8—C30	107.6 (3)	C26—C25—N7	130.1 (3)
C24—N8—H8A	126.2	C27—C26—C25	117.2 (4)
C30—N8—H8A	126.2	C27—C26—H26A	121.4
C32—N9—C33	106.2 (3)	C25—C26—H26A	121.4
C32—N9—Fe1	112.1 (2)	C26—C27—C28	121.4 (4)
C33—N9—Fe1	137.3 (3)	C26—C27—H27A	119.3
C32—N10—C38	107.7 (3)	C28—C27—H27A	119.3
C32—N10—H10A	126.2	C29—C28—C27	121.9 (4)
C38—N10—H10A	126.2	C29—C28—H28A	119.1
N1—C1—C2	114.9 (3)	C27—C28—H28A	119.1
N1—C1—C6	108.1 (3)	C28—C29—C30	117.0 (4)
C2—C1—C6	113.9 (3)	C28—C29—H29A	121.5
N1—C1—H1A	106.5	C30—C29—H29A	121.5
C2—C1—H1A	106.5	C29—C30—N8	132.9 (4)
C6—C1—H1A	106.5	C29—C30—C25	121.7 (4)
C3—C2—C1	112.9 (4)	N8—C30—C25	105.4 (3)
C3—C2—H2A	110 (3)	N2—C31—C32	110.9 (3)
C1—C2—H2A	108 (3)	N2—C31—H31A	109.5
C3—C2—H2B	110 (3)	C32—C31—H31A	109.5
C1—C2—H2B	110 (3)	N2—C31—H31B	109.5
H2A—C2—H2B	105 (4)	C32—C31—H31B	109.5
C4—C3—C2	110.6 (3)	H31A—C31—H31B	108.0
C4—C3—H3A	109.5	N9—C32—N10	112.4 (3)
C2—C3—H3A	109.5	N9—C32—C31	123.8 (3)
C4—C3—H3B	109.5	N10—C32—C31	123.9 (3)
C2—C3—H3B	109.5	C34—C33—C38	121.1 (3)
H3A—C3—H3B	108.1	C34—C33—N9	131.1 (3)
C3—C4—C5	109.9 (3)	C38—C33—N9	107.8 (3)
C3—C4—H4B	109.7	C33—C34—C35	117.1 (4)
C5—C4—H4B	109.7	C33—C34—H34A	121.4
C3—C4—H4C	109.7	C35—C34—H34A	121.4
C5—C4—H4C	109.7	C36—C35—C34	121.5 (4)
H4B—C4—H4C	108.2	C36—C35—H35A	119.2
C6—C5—C4	112.4 (3)	C34—C35—H35A	119.2
C6—C5—H5A	109.1	C37—C36—C35	122.3 (4)
C4—C5—H5A	109.1	C37—C36—H36A	118.9
C6—C5—H5B	109.1	C35—C36—H36A	118.9
C4—C5—H5B	109.1	C36—C37—C38	116.4 (4)
H5A—C5—H5B	107.8	C36—C37—H37A	121.8
N2—C6—C5	114.8 (3)	C38—C37—H37A	121.8
N2—C6—C1	108.7 (3)	N10—C38—C37	132.5 (4)
C5—C6—C1	114.2 (3)	N10—C38—C33	105.9 (3)
N2—C6—H6B	106.2	C37—C38—C33	121.5 (4)
C5—C6—H6B	106.2	O2—Cl1—O1	115.1 (4)
C1—C6—H6B	106.2	O2—Cl1—O3	107.8 (4)
C8—C7—N1	112.0 (3)	O1—Cl1—O3	106.4 (4)
C8—C7—H7A	109 (2)	O2—Cl1—O4	113.0 (3)
N1—C7—H7A	108 (2)	O1—Cl1—O4	106.9 (3)
C8—C7—H7B	110 (2)	O3—Cl1—O4	107.2 (3)
N1—C7—H7B	111 (2)	O8—Cl2—O6	101.0 (9)
H7A—C7—H7B	107 (3)	O8—Cl2—O5	126.2 (13)
N3—C8—N4	112.5 (3)	O6—Cl2—O5	102.8 (11)
N3—C8—C7	124.4 (3)	O8—Cl2—O7	108.3 (8)
N4—C8—C7	123.1 (3)	O6—Cl2—O7	115.9 (11)
C10—C9—N3	131.8 (4)	O5—Cl2—O7	103.3 (8)
C10—C9—C14	119.8 (3)	O5'—Cl2'—O7'	106.4 (14)
N3—C9—C14	108.4 (3)	O5'—Cl2'—O8'	112.4 (12)
C11—C10—C9	117.3 (4)	O7'—Cl2'—O8'	104.5 (11)
C11—C10—H10B	121.3	O5'—Cl2'—O6'	105.8 (12)
C9—C10—H10B	121.3	O7'—Cl2'—O6'	102.2 (10)
C10—C11—C12	121.7 (4)	O8'—Cl2'—O6'	123.9 (11)
C10—C11—H11A	119.1	C1S—O1S—H1S	107.9
C12—C11—H11A	119.1	O1S—C1S—H1SA	109.1
C13—C12—C11	122.3 (4)	O1S—C1S—H1SB	110.0
C13—C12—H12A	118.8	H1SA—C1S—H1SB	109.2
C11—C12—H12A	118.8	O1S—C1S—H1SC	110.7
C12—C13—C14	116.3 (4)	H1SA—C1S—H1SC	109.2
C12—C13—H13A	121.8	H1SB—C1S—H1SC	108.5

### Hydrogen-bond geometry (Å, °)

**Table:**

*D*—H···*A*	*D*—H	H···*A*	*D*···*A*	*D*—H···*A*
N4—H4A···O1^i^	0.86	2.10	2.952 (6)	171
N6—H6A···O6'^ii^	0.86	2.02	2.852 (15)	162
N6—H6A···O6^ii^	0.86	2.13	2.955 (16)	162
N8—H8A···O1S	0.86	1.94	2.790 (5)	170
N10—H10A···O7'^iii^	0.86	2.04	2.896 (10)	176
N10—H10A···O8^iii^	0.86	2.01	2.787 (12)	150
O1S—H1S···O3^iv^	0.96	1.88	2.813 (6)	166
C7—H7B···O4^i^	0.94 (4)	2.34 (4)	3.202 (6)	152 (3)
C23—H23A···O2^iv^	0.97	2.40	3.318 (6)	158

Symmetry codes: (i) −*x*+1, −*y*+1, −*z*; (ii) −*x*+1/2, *y*+1/2, −*z*+1/2; (iii) −*x*+1, −*y*+1, −*z*+1; (iv) −*x*+3/2, *y*+1/2, −*z*+1/2.
